# Research Agenda of Climate Change during and after the Coronavirus Disease 2019 (COVID-19) Pandemic

**DOI:** 10.3390/jcm10040770

**Published:** 2021-02-15

**Authors:** Hiroshi Nishiura, Nobuo Mimura

**Affiliations:** 1 Kyoto University School of Public Health, Yoshidakonoecho, Sakyoku, Kyoto City 606-8501, Japan; 2 Ibaraki University Global and Local Environment Co-creation Institute, Bunkyo 2-1-1, Mito City 310-8512, Japan; nobuo.mimura.iu@vc.ibaraki.ac.jp

## 1. Introduction

The global pandemic of coronavirus disease 2019 (COVID-19) rapidly spread worldwide during the first few months of 2020. Most industrialized countries had to adopt so-called “suppression strategies” against COVID-19, aiming to greatly reduce virus transmission and to limit deaths and the demand for critical care services. These suppression strategies have required substantial reductions in the rate of human-to-human contact, sometimes via legally binding countermeasures including “lockdown” policies in urban areas [1]. Once such countermeasures were in place, levels of human mobility decreased dramatically, resulting in an enormous reduction in commuting owing to work-from-home policies (i.e., remote or telework). Domestic and international travel as well as other means of transportation declined drastically. It is expected that widespread use of specific methods of COVID-19 prevention (e.g., vaccination) and treatment (e.g., combination therapy using antivirals and steroids) will begin soon; however, the virus that causes COVID-19, severe acute respiratory syndrome coronavirus 2 (SARS-CoV-2), is likely to continue circulating widely at least during the 2020/2021 winter season in the Northern Hemisphere.

Such drastic and artificial changes in societies around the world have provided an unprecedented opportunity for the scientific community to study climate change. Global air pollution and carbon dioxide (CO_2_) emissions were instantaneously reduced for a certain duration of time [2], offering a very important chance for the general public to experience improved air quality and reduced CO_2_ emissions levels. Nevertheless, the impact of COVID-19 on climate change comprises both positive and negative elements because many COVID-19 countermeasures have had severely negative economic impacts. Moreover, plans for mitigation as well as adaptation are also considerably affected by the “new normal” lifestyles. Under the direction of the Ministry of the Environment, Japan, the Environmental Restoration and Conservation Agency of Japan (ERCA) has launched a strategic financial support program, the Environment Research and Technology Development Fund, to support “Integrated Research on Climate Change Impact Assessment and Adaptation Plans” (S-18; project leader: Nobuo Mimura), beginning in the fiscal year 2020. The project aims to provide up-to-date scientific evidence to assist decision-making regarding governmental policies for adaptation planning that also address climate change. As life under the new normal conditions progresses worldwide, our strategic research program should also be flexibly set to measure the scientific features of the relationship between COVID-19 and climate change. In this editorial, we aim to discuss the research agenda regarding this particular relationship.

## 2. Impact of Climate Change on COVID-19

**Figure 1:**
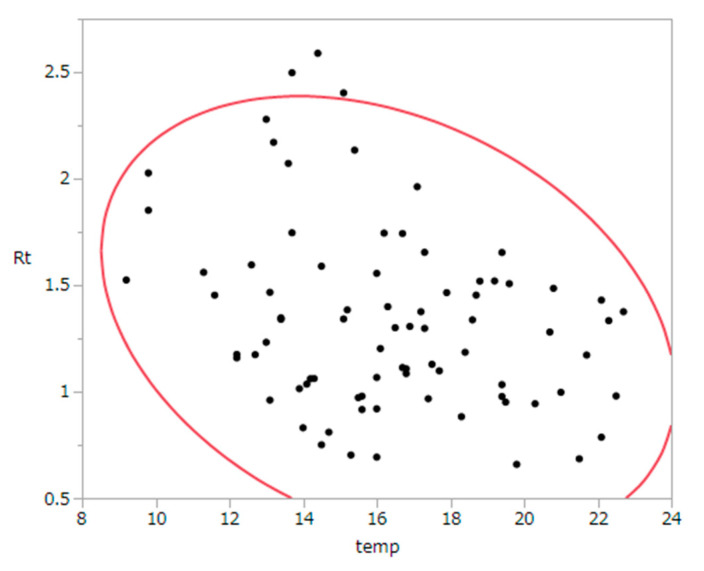
Bivariate relationship between the effective reproduction number and daily average temperature in October 2020, observed in four cities (Sapporo, Sendai, Tokyo, and Osaka) in Japan. No time lag between two variables was considered because the reproduction number reflects an instantaneous measure of transmission on a given day, on which temperature may have an effect.

Two issues must be debated regarding the impact of climate change on COVID-19. First, we have begun to understand how temperature levels could have a direct impact on human-to-human transmission of COVID-19 and how cold weather increases secondary transmission of SARS-CoV-2. At the moment, it is very likely that cold weather could increase the spread of COVID-19 [3]. This epidemiological finding is consistent with current experimental evidence indicating lower levels of transmission in warmer and more humid environments [4]. Countries in temperate zones have progressively experienced second epidemic waves during the summer season, with a continuous supply of individuals susceptible to infection with the novel coronavirus. Analyzing the confirmed case data in Japan during autumn 2020 in four urban cities (Sapporo, Miyagi, Tokyo, and Osaka), in the absence of interventions and estimating the effective reproduction number (i.e., the average number of secondary cases generated by a single primary case), we observed a clear negative correlation between the effective reproduction number and daily average temperature (*r* = −0.31, *p* < 0.01; Figure 1). As a possible mechanism of intensified transmission on cold days, a well-designed time allocation study in labor economics clarified that people are more likely to spend time in indoor spaces on cold days [5]. In addition to behavioral changes under cold environmental conditions, physical mechanisms could support a causal relationship. Although the abovementioned facts have gradually emerged as scientific evidence, it is reasonable to assume that the way in which changing weather conditions will affect the transmission of COVID-19 remains unclear. To control the spread of COVID-19 infections, we cannot solely rely on warmer weather to curtail transmission.

Second, more broadly, the COVID-19 pandemic offers a unique research opportunity to investigate the increasing frequency of emerging infectious diseases over time [6]. Most novel human pathogens have originated in animals, especially wild animals, and the risk of emergence is known to be dependent on the frequency of human exposure at the human–animal interface. To date, plausible explanations for the increasing frequency of novel pathogen emergence include (i) increased local and international travel, (ii) increased risk of exposure to wild animals that are likely to harbor novel human pathogens, and (iii) habitat loss of wild animals owing to expanded agricultural land use and urbanization. Climate change also acts as a possible cause of pathogen emergence because changes in the climate can induce species extinction, habitat changes, and migration of wild animals, such as toward polar regions to escape rising heat levels. However, such mechanisms largely remain under scientific debate, and objective demonstration of such phenomena has yet to be achieved in a bottom-up fashion. Were the relevance of climate change to be scientifically proven, many adaptation measures should be considered to reduce the likelihood of the emergence of further human pathogens with pandemic potential.

## 3. Positive and Negative Impacts of COVID-19 on Climate Change

Reverse causal effects (e.g., complications in climate change) during the aftermath of the COVID-19 pandemic have yielded both positive and negative impacts. A critically important positive impact is the resulting reduced levels of CO_2_ emissions during 2020, which represents a crucial moment in terms of countermeasures that can mitigate climate change. The global population has directly experienced reduced CO_2_ emissions and improved air quality. However, decreases in CO_2_ emissions owing to the COVID-19 pandemic account for only a small proportion of the planned reduction target [7], requiring that populations develop greater acceptance of radical countermeasures. Of course, mitigation plans against global warming that offer unique solutions aiming to further reduce CO_2_ emissions should be encouraged. Additionally, the post-pandemic period presents an important opportunity to implement adaptation measures, to recover public trust. People are now better able to understand the seriousness of global warming; therefore, preparedness against the adverse impacts of climate change can be strengthened during this window of opportunity.

There are numerous possible negative impacts of the COVID-19 pandemic on climate change. Importantly, we must remember that people with underlying comorbidities and those with lower incomes are disproportionately affected by both COVID-19 and climate change. Thus, the physical impact of the COVID-19 pandemic together with climate change is more intense in socioeconomically underdeveloped countries and populations. Global society should protect these vulnerable populations from this double burden by maintaining solidarity with these countries. 

The financial impact of COVID-19 on climate change has been huge, and it has become clear that the global economy is not very resilient. This is bad news for continuing and additionally implementing responses to climate change because these include many costly measures, such as transformation to renewable energy sources, electrification of transportation systems, and other clean technologies. At minimum, policy leaders in industrialized nations should aim to take action toward climate-resilient recovery during the post-pandemic period. 

It is also vital to remember that social changes owing to COVID-19 can lead to complications with respect to future adaptation. For instance, the time and frequency that care workers spend with elderly people has been greatly reduced, to avoid unnecessary close contact in confined spaces. However, this situation can lead to lower levels of care and monitoring in elderly populations aimed to avoid problems such as heat stroke, where older people may need to be reminded to drink water and use air conditioning. Such critical points should be thoroughly explored over the course of the pandemic.

## 4. The Way Forward

We have briefly described the research agenda surrounding the COVID-19 pandemic in terms of climate change. Owing to the pandemic, climate change studies in 2020 have partly stagnated with researchers forced to work remotely. However, taking advantage of the heightened awareness of the mutual impact of the COVID-19 pandemic and climate change, important modifications have taken place owing to physical, financial, and social conditions being altered during the pandemic. Concerted research efforts on this subject are called for.
